# The Social Modulation of Pain: Others as Predictive Signals of Salience – a Systematic Review

**DOI:** 10.3389/fnhum.2013.00386

**Published:** 2013-07-23

**Authors:** Charlotte Krahé, Anne Springer, John A. Weinman, Aikaterini Fotopoulou

**Affiliations:** ^1^Department of Psychology, Institute of Psychiatry, King’s College London, London, UK; ^2^Department of Sport and Exercise Psychology, University of Potsdam, Potsdam, Germany; ^3^Research Department of Clinical, Educational and Health Psychology, University College London, London, UK

**Keywords:** pain, social modulation, social support, empathy, predictive coding, attachment, review

## Abstract

Several studies in cognitive neuroscience have investigated the cognitive and affective modulation of pain. By contrast, fewer studies have focused on the social modulation of pain, despite a plethora of relevant clinical findings. Here we present the first review of experimental studies addressing how interpersonal factors, such as the presence, behavior, and spatial proximity of an observer, modulate pain. Based on a systematic literature search, we identified 26 studies on experimentally induced pain that manipulated different interpersonal variables and measured behavioral, physiological, and neural pain-related responses. We observed that the modulation of pain by interpersonal factors depended on (1) the degree to which the social partners were active or were perceived by the participants to possess possibility for action; (2) the degree to which participants could perceive the specific intentions of the social partners; (3) the type of pre-existing relationship between the social partner and the person in pain, and lastly, (4) individual differences in relating to others and coping styles. Based on these findings, we propose that the modulation of pain by social factors can be fruitfully understood in relation to a recent predictive coding model, the free energy framework, particularly as applied to interoception and social cognition. Specifically, we argue that interpersonal interactions during pain may function as social, predictive signals of contextual threat or safety and as such influence the salience of noxious stimuli. The perception of such interpersonal interactions may in turn depend on (a) prior beliefs about interpersonal relating and (b) the certainty or precision by which an interpersonal interaction may predict environmental threat or safety.

## Introduction

Pain is a subjective psychological state which acts as an indicator of threat to the organism in association with actual or potential tissue damage (IASP, 1994). Pain is multidimensional, including unpleasant feelings (interoception; Craig, 2002) and sensations (nociception) about the state of the organism, as well as motivated behaviors, such as withdrawing from a noxious stimulus. Several studies in cognitive neuroscience have investigated the cognitive and affective modulation of pain (e.g., Tracey et al., 2002). For example, attention (e.g., Villemure and Bushnell, 2002), mood (e.g., Yoshino et al., 2010), and cognitive appraisals (e.g., Vlaeyen et al., 2009) have been found to modulate pain. By contrast, the modulation of pain by social factors has received far less experimental and neuroscientific attention to date. This is despite a plethora of clinical, correlational findings pointing to associations between pain and the social context in which it occurs (Leonard et al., 2006).

Close relationships are beneficial to both mental and physical health, including stress and pain. For example, a wealth of research has shown that support from others is linked to beneficial effects on physiological and psychological well-being (Uchino et al., 1996; Blasi et al., 2001; Kikusui et al., 2006), while social isolation and poor quality relationships are detrimental to health (House et al., 1988). However, support from others is not a panacea; rather, the effects of social support on health, such as stress and pain, depend on the facet of social support studied (e.g., Schaefer et al., 1981; Barrera, 1986) and on factors such as gender or relationship characteristics (Kirschbaum et al., 1995; Hennessy et al., 2009).

A similar picture emerges when studying social influences on pain. While there has been much research in clinical populations (e.g., Penner et al., 2008; Williams et al., 2011) and chronic pain populations more specifically (see below for a brief review), fewer studies have experimentally investigated the role of social context on pain in healthy individuals. Although clinical pain differs from experimentally induced pain (McGrath, 1983), studies in the latter tradition are indispensable for elucidating causal influencing factors because they allow controlled manipulation of the social variables of interest. Interestingly, such experimental manipulations reveal that multidimensional concepts such as social support may not be sufficient to characterize the social modulation of pain. Rather, particular facets of the social context seem to differentially influence whether and how interpersonal interactions can affect pain. Thus, this diversity and the specific causal mechanisms by which different social factors influence pain warrant systematic consideration. Unfortunately, to the best of our knowledge, there is no systematic review of these studies. Accordingly, the present paper aimed to provide a systematic review of studies that experimentally investigated the effects of interpersonal factors on pain with a focus on discovering the underlying causal mechanisms.

In addition, we aimed to use a framework from computational neuroscience, namely the free energy framework as applied to interoception (Seth et al., 2012) and social cognition (Brown and Brüne, 2012), as the theoretical basis for the integration and understanding of the findings presented in this review. Several perspectives exist which view pain (Sullivan et al., 2001; Craig, 2009a) and emotion more generally (Griffiths and Scarantino, 2009; Van Kleef, 2009; Coan, 2011) as embedded within a social context and posit mechanisms by which social partners affect an individual’s experience (e.g., by providing support or contextual information). Adding to these, we believe a predictive coding scheme, such as the free energy framework (see below), to be particularly promising because it provides a unifying, neurobiologically plausible account of the integration of different hierarchical levels of processing, from nociception to social cognition. It can thus shed light on the mechanisms by which interpersonal factors affect pain-related perceptions and actions. Furthermore, this framework places emphasis on how pre-existing mental models shape current perception and action at different time scales. This focus is consistent with the pain literature under consideration, which has long stressed the pivotal role influence of anticipatory cognitions and emotions on pain (e.g., Wiech et al., 2010), as well as the corresponding social literature that has underlined the role of pre-learned social relating schemas in subsequent perceptions and reactions (Meredith et al., 2006).

Bayesian predictive coding models such as the free energy framework are powerful theoretical and neurobiological models of perception and action (Dayan and Hinton, 1996; Rao and Ballard, 1999; Schultz and Dickinson, 2000). The essence of these models is that neurobiological message-passing in the brain is achieved by coding potentially ambiguous (noisy) incoming information in light of prior expectations about the likely sensory causes of such information. Further, the related hypotheses (“generative models”) of the hidden causes of sensory input are constantly updated on the basis of mismatches between expectation and experience (“prediction errors,” also conceptualized as free energy), and optimized so as to minimize prediction error. While the above describes perceptual inference, the free energy framework includes a parallel process of active inference, which entails acting on the environment to change sensory input and also leads to the optimization of prediction errors (Friston et al., 2012). In general terms, prediction errors are assumed to be conveyed by feed-forward connections from lower to higher neural levels to improve representations in the latter, and higher-order predictions are transferred via feedback connections that can suppress prediction errors in lower levels. The reciprocal but asymmetric characteristics of this hierarchy (see also Mesulam, 2012) allow for an optimization that makes every level accountable to the others, delivering an internally consistent re-representation of sensory causes at multiple levels of the neurocognitive hierarchy.

Unfortunately, most psychological models based on the free energy framework concern exteroception (the perception of the environment or the self via, e.g., vision and hearing) and proprioception (the sense of the position of the body in space). Only very recently, a predictive coding model of interoceptive awareness has been proposed, describing subjective feeling states as arising from predictive inferences on the causes of interoceptive signals (Seth et al., 2012). With regard to pain, such a model is highly relevant because pain has recently been re-classified as an interoceptive modality (Craig, 2002, 2009b). Interoception in this renewed sense does not refer only to visceral sensation but to the central processing of all homeostatic afferent activity that can reflect the various components of the physiological condition of the body. In this view, pain and all feelings from the body are processed peripherally and centrally by a recently discovered lamina I spinothalamocortical pathway that projects to the posterior granular and mid-dysgranular regions of the insular cortex (serving as primary interoceptive cortex) via the brainstem parabrachial nucleus and posterior part of the ventromedial thalamic nuclei (Craig, 2003, 2009b). Primary interoceptive signals are thought to be represented in the mid/posterior insula, where they are also integrated with exteroceptive information coming from different brain areas. Further re-mappings within the anterior insula, the anterior cingulate cortex (ACC), and the orbitofrontal cortex are thought to consolidate body-state signals with social, motivational, and contextual information to ultimately give rise to the conscious experience of emotions, as well as to prepare the organism for the necessary action in the environment (Damasio et al., 2000; Craig, 2002, 2009b; Critchley, 2005).

A number of recent neuroimaging studies have included such areas and their observed functional connectivity in various hypothesized “salience networks” (Seeley et al., 2007; Medford and Critchley, 2010; Wiech et al., 2010; Legrain et al., 2011; Cauda et al., 2012). For instance, predictive signals from such a “salience network” process and integrate information about the significance of an impending noxious stimulus and determine whether or not such a stimulus will be consciously perceived as painful (Wiech et al., 2010), and indeed the insula cortex responds to interoceptive stimuli on the basis of expectations (Seth et al., 2012). Thus, the neural regions involved in interoception generate predictive signals of interoceptive salience. Pain can therefore constitute a process of perceptual inference about nociceptive signals on the basis of predictive, top-down signals about the homeostatic significance of such signals in the context of other synchronous biological, cognitive, and social conditions.

Furthermore, such re-mappings of interoceptive signals across the neurocognitive hierarchy suggest possible neurobiological mechanisms by which not only cognitive, but also social contextual factors can influence the awareness of interoceptive and other multimodal information about one’s own body. In pain research, it is established that nociception (“the neural process of encoding noxious stimuli”; IASP, 1994) is not sufficient to explain the conscious experience of pain (e.g., Hofbauer et al., 2004; Baumgärtner et al., 2006; Nikolajsen and Jensen, 2006; Lee et al., 2009), and it has been repeatedly demonstrated that psychosocial factors can have important top-down effects on pain (e.g., the studies discussed in the present review). Thus, the application of the free energy framework to pain may be particularly fruitful to generate organized accounts of the dynamic relations between bottom-up (e.g., nociception) and top-down (e.g., psychosocial) influences on pain. In addition, pain engenders action, e.g., it motivates behaviors designed to ensure the organism is no longer under threat (Auvray et al., 2010; Wiech and Tracey, 2013). Hence, perceptual and motivational aspects of pain can be unified under the same optimization principle within a free energy framework. In particular, the motivational aspects of pain can be conceptualized as a process of active inference, where actions are performed to change nociceptive input and update predictions. In a social context, such actions may elicit help from others and change sensations via this social channel.

In the following, we explain the inclusion criteria and methods applied to our review (see Method), present the results (see Results and their Organization) and place these findings in the broader context of the free energy framework introduced above (see Discussion) to illustrate how interpersonal interactions may be integrated at different neural levels to influence the perception of pain and related behavioral responses. Before turning to these sections, we briefly consider two other research traditions, namely studies on the social modulation of clinical chronic pain, and pain in animals. While these traditions fall outside the remit of this review, we consider it important to briefly summarize their main findings as an introduction to the potential psychological and neurobiological mechanisms that may mediate the social modulation of pain in healthy human populations (see also, e.g., Payne and Norfleet, 1986; Newton-John, 2002; Cano, 2004; Panksepp, 2006; Cano et al., 2008; Mogil, 2009, respectively).

### Insights from clinical studies

In clinical pain populations, a wealth of research has focused on the role of social support in chronic pain, and on the relationship between the pain patient and their partner (e.g., Block, 1981; Flor et al., 1987; Boothby et al., 2004; Cano et al., 2005). While some studies report correlations between perceived social support and lower pain intensity (López-Martínez et al., 2008), others have found a positive association between social support and pain behaviors (e.g., Gil et al., 1987), level of pain (Flor et al., 1987; Kerns et al., 1990), and disability (Romano et al., 1995). The majority of research has drawn on behavioral models to explain these associations, focusing strongly on operant conditioning (Cano and Williams, 2010). The operant conditioning perspective posits that repeated instances of social support serve to reward or punish pain behaviors, leading to positive or negative reinforcement of such behaviors. While this model has been broadly supported, also in an experimental study (Jolliffe and Nicholas, 2004), it does not include cognitive and affective factors and thus may not offer a complete picture of the complexity of social interactions (Newton-John, 2002). Cognitive-behavioral models focusing more on pain appraisals have emerged. One prominent example is the communal coping model of pain catastrophizing (e.g., Sullivan et al., 2001), which claims that individuals who tend to catastrophize – that is, exaggerate the threat value of pain and see themselves as unable to cope with pain themselves (Keefe et al., 2000) – might engage in more pain behaviors to attract support from others. Here, pain appraisals play a key role in the social context of pain. A further perspective integrating cognitive factors and placing them within a relationship context is the intimacy model (see Cano and Williams, 2010), in which communicating pain to a partner is viewed as an attempt to create and maintain an emotionally intimate relationship environment.

In sum, clinical pain studies, although correlational in nature, have led to the development of several models which have been adapted to experimental settings (e.g., Sullivan et al., 2004). Furthermore, clinical studies investigate long-term pain, in which pain appraisals may be more strongly established than in the transient context of experimental settings. As many chronic pain studies focus on the partner as supportive other, they also address the importance of the relationship between supportive other and pain patient. Thus, their findings are important in fostering our understanding of psychological mechanisms underlying the social modulation of pain in humans.

### Insights from animal studies and implications for human research

Although direct comparisons between human and animal studies are not warranted, animal studies can provide tentative neurobiological insights into the social modulation of pain. Animals and particularly mammals are highly sociable, and many animals – including humans – rely on parental care for survival in early life. To regulate proximity to these critical caregivers, animals and humans possess an attachment system which manifests itself in the formation of close social bonds (Panksepp, 1998). Several studies have investigated whether such social bonds influence pain in animals, typically by studying the behavior of mouse dyads while pain is induced in one dyad member. Langford and colleagues found that female mice approaching a dyad member in pain led to less writhing from the mouse in pain. Crucially, these beneficial effects of social contact were seen only when the approaching mouse was a cagemate of the mouse in pain rather than a stranger (Langford et al., 2010). In this vein, D’Amato and Pavone (1993) discovered that interacting with siblings reduced pain sensitivity in mice, whilst interacting with stranger mice did not. In addition to establishing that close social relationships modulate pain in mice, these studies have also shed light on possible underlying neurobiological mechanisms. Specifically, endogenous opioids and oxytocin have been implicated, the former relating to reinforcement of social emotions (D’Amato and Pavone, 1993) and the latter playing an important role in social bonding (for a review, see Campbell, 2010). Regarding endogenous opioids, D’Amato and Pavone found that their socially induced analgesic effects were blocked when mice received naloxone, an opioid antagonist, pointing to a mediating role of endogenous opioids. Oxytocin has been linked to pain reduction *per se* (Yu et al., 2003) and interacts with opioid and also dopaminergic systems, with dopamine driving the motivation to affiliate and form social bonds (McCall and Singer, 2012). Furthermore, oxytocin exerts positive effects such as preventing the development of depressive-like behavior in socially isolated mice with nerve damage (Norman et al., 2010). Therefore, these proposed neurobiological mechanisms seem to relate both to social bonding and pain.

Indeed, similarities between pain and social loss have been observed in animals (Panksepp et al., 1997): both pain and social experiences include threat, unpleasantness, and loss (e.g., of a function or fellow animal) in phenomenological terms, and from a neurological viewpoint, opioid administration seems to alleviate both bodily pain and the pain of social isolation/absence of a caregiver. In light of these similarities, Panksepp and colleagues proposed that the drive to seek proximity and avoid separation is built upon the foundations of the older pain system (including, e.g., the opioid system); thus, social loss or separation hurts. Though caution is necessary when making comparisons between animals and humans, the neural links between pain and the distress of social loss have been investigated in humans.

In humans, higher baseline pain sensitivity has been linked to greater distress following social rejection, and heightened levels of social distress have been associated with more pain unpleasantness on thermal pain induction following social rejection (Eisenberger et al., 2006). Functional neuroimaging studies have suggested that similar neural regions to those implicated in bodily pain are activated during social pain (e.g., the dorsal ACC; Eisenberger et al., 2003; Eisenberger, 2012) though whether these regions are pain-specific is debated (e.g., Legrain et al., 2011; Mouraux et al., 2011). Taken together, these studies propose tentative neural mechanisms involved in the social modulation of pain and social connection in animals and as well as humans and underline the importance of close attachment bonds.

## Method

### Selection of studies

We conducted a systematic search of the on-line databases Web of Knowledge, PubMed, PsycInfo, and Google Scholar, using combinations of the following keywords: “pain,” “interpersonal,” “empathy,” “attachment,” “social context,” “social interaction,” “social support,” “social presence,” and “social modulation.” Reference lists of relevant articles were also searched. The results were assessed for inclusion using the publication title and abstract. No restrictions regarding publication dates were applied.

Studies were included if they conformed to the following five *a priori* inclusion and exclusion criteria. Firstly, we excluded wider societal (intergroup) influences. Naturally, pain is generally experienced within the wider social world, and gender (e.g., Levine and De Simone, 1991), ethnicity (e.g., Weisse et al., 2005), and in-group/out-group influences (Buss and Portnoy, 1967), to name but a few, undoubtedly contribute to the social modulation of pain. However, we focused our review on experimental studies examining interpersonal influences rather than the larger social context of pain to advance our understanding of the causal interpersonal mechanisms that may shape an individual’s pain experience. Specifically, we included those studies which manipulated an embodied or primed interpersonal exchange “between two or more individuals which is very largely determined by their individual characteristics and the nature of the personal relations between them” (the interpersonal extreme, Tajfel, 1982, p.13) and excluded “interactions which are largely determined by group memberships of the participants and very little – if at all – by their personal relations or individual characteristics” (the intergroup extreme, Tajfel, 1982, p.13). Hence, we excluded studies that varied, for example, experimenter gender or race, unless they also manipulated aspects of an interpersonal interaction. We also excluded social modeling studies in which *both* interaction partners received pain (or were made to believe the other received pain). Consistent with the tradition of studies addressing social support, our focus was on how the person in pain was affected by interpersonal interactions with pain-free individuals.

Secondly, studies were included if they experimentally induced pain (e.g., by means of a coldpressor task) and excluded if they used clinical procedures such as routine immunizations. We excluded clinical procedures because they differed from laboratory studies in the use of health-related procedures (which implicate additional variables such as illness perceptions, medical history etc.) and in the degree of experimental control (for a discussion, see Manimala et al., 2000). Thirdly, related to the previous point, studies were included if they examined the *causal* effects of interpersonal interactions on pain and were excluded if they merely correlated pain data with social variables. Fourthly, studies were included if they reported behavioral pain outcomes (e.g., pain intensity ratings, facial expressions) or pain-related physiological outcomes. To render studies comparable, neuroimaging studies were only included if they also yielded behavioral or physiological data. Finally, studies were included if they were published in English.

## Results and their Organization

Twenty-six studies met the above five inclusion criteria. A summary of included studies is presented in Table 1. The terms “participant” and “person in pain” refer to the individual receiving pain, while “social partner” denotes the individual interacting with the person in pain, e.g., providing support. A variety of terms have been used in the literature in relation to the social modulation of pain, including “social support,” “social interaction,” “interpersonal interaction,” “social presence,” “social influence,” and “social context.” Each of these terms appears to place a slightly different emphasis on the social partner; for example, “social support” implies a directly caring attitude toward the person in pain compared to the broader term “social context.” For the purposes of our analysis, we used the term “social context” when discussing the role of others generally and “interpersonal interaction” when discussing specific interactions between social partner and participant in pain as outlined above.

**Table 1:** **Effects of interpersonal interactions on pain: a summary of experimental studies to date**.

Reference	Social manipulation(s)	Sample	Social partner	Pain induction technique	Pain measures	Findings
Borsook and MacDonald (2010)	Between-subjects design: 1) Alone 2) Positive encounter 3) Negative encounter after baseline pain induction	Healthy students (*N* = 45)	Stranger	Pressure pain	Pain ratings	The negative encounter condition reduced pain intensity and unpleasantness relative to baseline. The other two conditions showed no change
Brown et al. (2003)	Between-subjects design: 1) Alone 2) Passive support (physical presence) 3) Active support 4) Interaction during pain	Healthy students (*N* = 101)	Friend or stranger	Coldpressor	Pain ratings	Participants in active and passive support conditions felt less pain than participants in alone or interaction conditions, regardless of type of interaction partner
Chambers et al. (2002)	Between-subjects design: 1) Pain-promoting (reassurance, empathy) 2) Pain-reducing (distraction) 3) Neutral interaction (interact as normal) during pain	Healthy children aged 8–12 (*N* = 120)	Mother	Coldpressor	Pain ratings; pain behaviors; physiological measures	For girls, pain intensity was highest in the pain-promoting condition, followed by neutral interaction and pain-reducing conditions. No differences in pain intensity between conditions were found for boys. Maternal interaction type did not affect other pain measures
Eisenberger et al. (2011)	Within-subjects design: Viewing pictures during pain	Healthy females in a relationship (*N* = 17)	Romantic partner, stranger, and object	Thermal pain	Pain ratings; neural activity	Pain ratings were lower when viewing partner pictures than when viewing stranger and object pictures. Participants showed less activity in the dACC and bilateral anterior insula when viewing partner pictures vs. other two conditions on high pain trials
Flor et al. (1995)	Within-subjects design: 1) Partner present 2) Partner absent during pain and 1) Neutral interaction 2) Conflict interaction after pain inductions 1 and 2, respectively	Chronic back pain patients (*n* = 17) and healthy controls (*n* = 15)	Romantic partner	Coldpressor	Pain ratings; pain behaviors; physiological measures; pain words	Patients with highly solicitous partners showed lower pain threshold and tolerance when the partner was present vs. absent. This was not found for patients with non-solicitous partners
Hayes and Wolf (1984)	Between-subjects design: 1) Coping privately 2) Coping publicly 3) Attention-placebo control group between pain inductions	Students (*N* = 84)	Stranger (experimenter)	Coldpressor	Pain ratings; pain behaviors	Participants in the coping publicly condition showed greater tolerance than participants in the control group. The private coping condition did not differ from control group
Jackson et al. (2005)	Study 1: Between-subjects design: 1) No transaction (NT) 2) Transaction opportunity (TO) during pain	Young healthy adults (*N* = 91)	Stranger (empathic experimenter)	Coldpressor	Pain ratings; pain behaviors	No differences between conditions and no condition by gender interaction were found. Within the TO condition, speaking to the experimenter was related to lower pain tolerance, higher pain intensity, and more catastrophizing than not speaking to the experimenter
	Study 2: Between-subjects design: 1) No transaction (NT) 2) Transaction opportunity (TO) 3) Distraction (DT) 4) Reinterpretation (RT) 5) Encouragement (ET) during pain	Healthy participants (N = 126)				Women showed higher pain tolerance in DT, RT, and ET than in NT and TO conditions. Lowest tolerance was shown in the TO condition. No differences across conditions were found for men
Jackson (2007)	Between-subjects design: 1) No transaction (NT) 2) Distraction 3) Pain monitoring 4) Reinterpretation during pain	Healthy participants (*N* = 118)	Female stranger	Coldpressor	Pain ratings; pain behaviors	Women displayed lowest tolerance in the NT condition vs. all other conditions. Women showed highest tolerance in the reinterpretation group, followed by pain monitoring, distraction, and NT conditions. Women’s reported pain intensity was highest in distraction condition, followed by NT, pain monitoring, and reinterpretation. No differences across conditions and measures were found for men
Jackson et al. (2009)	Between-subjects design: 1) Reassuring appraisal (participant and social partner read safety message) 2) Threat appraisal (both read threat message) 3) Mixed appraisal (participants read reassuring text, social partner read threatening text) before pain	Healthy participants (*N* = 86)	Person at least acquainted with (could include acquaintance, friend, partner)	Coldpressor	Pain ratings; pain behaviors; pain words used in dyad interactions during pain	Pain tolerance was reduced in the threat appraisal condition vs. reassuring and mixed appraisal conditions. Dyads in the threat appraisal condition used proportionally more pain words in conversation than reassured or mixed dyads. Mixed gender dyads used a higher proportion of pain words than same-sex dyads. Attention diversion by social partners increased pain tolerance
	During pain, social partners were present and helped participants cope in any way they chose	
Jolliffe and Nicholas (2004)	Between-subjects design: 1) Reinforcement 2) Non-reinforcement during pain	Healthy participants (*N* = 46)	Stranger	Pressure pain	Pain ratings; physiological measures	Participants in the reinforcement condition reported more pain than participants who received no reinforcement
Kleck et al. (1976)	Study 1: Within-subjects design: 1) Presumed alone 2) Observer present (behind one-way window) during pain	Healthy male participants (*N* = 20)	Stranger (higher-status female)	Electric shock	Pain ratings; pain behaviors; physiological measures	Participants were less expressive and had lower skin conductance levels when observed than when alone, especially in high shock trials. The magnitude of both these effects increased as shock intensity increased. Pain intensity was lower across shock levels in observer condition than when alone
	Study 2: Within-subjects design: 1) Alone 2) Male observer present 3) Female observer present during pain	Healthy male participants (*N* = 40)	Stranger (age peer stranger)		Pain ratings; pain behaviors; physiological measures	Participants expressed less discomfort, had lower skin conductance levels and reported less pain when observed than when alone, regardless of observer gender
Master et al. (2009)	Within-subjects design: 1) Holding hand/object 2) Viewing photographs during pain	Healthy females in a long-term relationship (*N* = 25)	Romantic partner and stranger and object	Thermal pain	Pain ratings	For hand-holding, women reported less pain unpleasantness when holding partner’s hand than when holding a stranger’s hand or an object. The same pattern of effects was observed in the photograph conditions. Effects of condition were not confounded with distraction
McClelland and McCubbin (2008)	Between-subjects design: 1) Alone 2) Physical presence during pain	Healthy students (*N* = 68)	Same-sex friend	Coldpressor	Pain ratings; physiological measures	Men reported less pain and women reported more pain in the presence vs. alone condition. Women, but not men, reported greater VAS pain in the presence condition than when alone. Women reported more affective and sensory pain than men in the presence condition. Participants with low support reported more pain in the alone condition than the presence condition; the opposite was found for high support participants. Participants in the presence condition had greater blood pressure changes than in the alone condition
Modic Stanke and Ivanec (2010)	Mixed design: Within-subjects factor: 1) Social presence 2) Absence during pain	Healthy females (*N* = 48)	Female stranger	Hot air	Pain ratings; pain behaviors	No significant effects of presence condition, distance condition, or their interaction
	Between-subjects factor: 1) Small physical distance (0.5 m) 2) Large physical distance (1.5 m) during pain	
Montoya et al. (2004)	Between-subjects design: 1) Alone 2) Physical presence during pain	Fibromyalgia patients (*n* = 18) and migraine patients (control group; *n* = 18)	Romantic partner	Thermal pain (hot and cold)	Pain ratings; neural activity	Fibromyalgia patients reported less pain sensitivity, higher pain threshold, lower pain ratings and reduced brain activity when partner present than when alone for heat but not cold pain. These effects were not observed in migraine patients
Peeters and Vlaeyen (2011)	Between-subjects design: 1) Low social threat 2) Neutral 3) High social threat during pain	Healthy participants (*N* = 82)	Stranger	Electrical pain	Pain ratings; pain behaviors	Increased social threat increased pain intensity ratings for high – but not low – pain catastrophizers; higher social threat reduced facial expression across participants
Platow et al. (2007)	Between-subjects design: 1) Reassurance from in-group member 2) Reassurance from out-group member 3) No reassurance between pain inductions	Healthy students (*N* = 54)	Stranger (in-group: same university degree; out-group: different university degree)	Coldpressor	Pain behaviors; physiological measures	No significant results for pain tolerance. Galvanic skin response during the second coldpressor trial was significantly lower in the in-group condition than in either out-group or no reassurance conditions. Physiological arousal was lower in high vs. low identifiers in the in-group condition
Sambo et al. (2010)	Within-subjects design: 1) Alone 2) Presence of high empathy observer 3) Presence of low empathy observer during pain	Healthy participants (*N* = 30)	Stranger	Thermal pain	Pain ratings; physiological measures	Skin conductance and heart rate were lower in both observer present conditions vs. being alone. Higher attachment anxiety predicted lower pain rating when high-empathic vs. low-empathic observer was present. Higher scores on attachment avoidance predicted lower pain ratings in alone vs. presence conditions
Sullivan et al. (2004)	Between-subjects design: 1) Alone 2) Observer present during pain	Healthy students (*N* = 64)	Stranger	Coldpressor	Pain ratings; pain behaviors	High catastrophizers showed communicative pain behaviors for a longer duration when observer present than when alone. This effect was not found for low catastrophizers. Within the presence condition, high catastrophizers reported more pain after the trial than low catastrophizers
Vervoort et al. (2008)	Between-subjects design: 1) Presence of parent observer 2) Presence of stranger observer during pain (children could not see observer)	Healthy children aged 9–15 years (*N* = 84)	Parent or stranger	Pressure pain	Pain ratings; pain behaviors	Low-catastrophizing children expressed less pain when stranger present vs. when parent present. High catastrophizing children expressed same amount of pain regardless of whether parent or stranger was observing. This pattern was not found for pain intensity or anxiety
Vervoort et al. (2011)	Within-subjects design: 1) Presumed alone (told no one observing but parent was observing from next room) 2) 3-min interaction with parent between CPT trials 3) Parent observing from next room during pain	Healthy children aged 10–18 years (*N* = 38)	Parent	Coldpressor	Pain ratings; pain behaviors	Low catastrophizers displayed more pain when observed than when presumed alone. High catastrophizing children showed same amount of pain when believed alone as when observed. Regardless of catastrophizing, pain intensity was lower when parent observed than when presumed alone. Higher levels of parental non-pain talk were related to increased facial expression and self-reports of pain only for high catastrophizing children. For low-catastrophizing children, both measures of pain were independent of parental pain talk
Vlaeyen et al. (2009)	Between-subjects design: 1) No threat/no observer 2) No threat/observer present 3) Threat/no observer present 4) Threat/observer present during pain	Healthy participants (*N* = 149)	Stranger	Coldpressor	Pain ratings; pain behaviors	Participants in the threat condition experiencing pain alone reported more pain than participants in the other three conditions. In the threatening context, the presence of a stranger inhibited pain facial expression during the pain induction. In the no threat conditions after the task, participants with an observer present displayed more pain expressions compared to participants who were alone
Wilson and Ruben (2011)	Within-subjects design: Interaction with partner before and during pain	Healthy couples – women took part in pain induction (*N* = 65)	Romantic partner	Muscle pain	Pain ratings; pain behaviors; physiological measures	Dismissively avoidant women showed lower pain tolerance and threshold when partner scored higher in attachment anxiety, and higher pain tolerance when partner scored lower in attachment anxiety (though pain intensity and physiological measures were not affected); secure women showed opposite pattern. Highest pain was reported when both couple members scored higher in attachment anxiety. Partner avoidance levels did not influence women’s pain
Younger et al. (2010)	Within-subjects design: 1) Viewing photographs 2) Distraction task (word-association task) during pain	Healthy students in early stages of romantic relationship (*N* = 15)	Romantic partner and acquaintance	Thermal pain	Pain ratings; neural activity	Partner task and distraction task both reduced pain, contrasted with viewing acquaintance photographs. No difference was found between partner task and distraction task. Pain relief in the partner condition was positively related to activation in the bilateral caudate head, bilateral nucleus accumbens, right dorsolateral prefrontal cortex, right superior temporal gyrus, bilateral lateral orbitofrontal cortex, left amygdala, and right thalamus (ventral anterior nucleus), and negatively related to activity in the right superior frontal gyrus, left dorsal anterior cingulate cortex, right brainstem, left anterior insula, right putamen, left supplementary motor area, and left parahippocampal area

Due to the large variety in pain measures obtained across studies, we summarized and presented pain measures in five sub-categories in the table. (1) “Pain ratings” refers to participant-generated ratings, e.g., of pain intensity, unpleasantness, or pain threshold; (2) “pain behaviors” denotes pain-related behaviors such as pain tolerance or facial expressions; (3) “pain words” refers to pain-related verbalizations to the social partner; (4) “physiological measures” pertains to measures of heart rate, skin conductance levels, blood pressure, etc.; and (5) “neural activity” signifies magnetoencephalography (MEG), and blood-oxygen-level-dependent (BOLD) measures obtained from functional magnetic resonance imaging (fMRI). Further, only findings relating to the above pain outcome measures were included in the table and additional variables such as gender or catastrophizing were included only if they interacted with the social manipulation in affecting pain. We address methodological issues where relevant in the results section.

The studies meeting the inclusion criteria were based in different research and theoretical traditions (e.g., health sciences, social psychology, clinical psychology, and social cognitive neuroscience). To ensure valid comparisons between studies, the theoretical context of such concepts, their taxonomy, and precise operationalization in each study were addressed when appropriate in the following sections, and they were taken into account when reviewing and integrating the data.

Moreover, the studies manipulated a variety of interpersonal factors including verbal interactions (e.g., Chambers et al., 2002; Jackson et al., 2005), non-verbal interactions (e.g., hand-holding; Master et al., 2009), mere physical presence of the social partner (Brown et al., 2003; Vervoort et al., 2008), priming by photographs of partners (e.g., Eisenberger et al., 2011) and manipulations of participants’ perception of the social partner (e.g., Sambo et al., 2010; Peeters and Vlaeyen, 2011). Also, studies differed in terms of the characteristics of their sample, for instance participant and partner gender, and personality traits (e.g., pain catastrophizing; Sullivan et al., 2004; or attachment style; Sambo et al., 2010; Wilson and Ruben, 2011). To review such heterogeneous data, and motivated by interactionist accounts of social cognition (e.g., Bartz et al., 2011), we drew a distinction between studies in which social partners were perceived by the participants to be active or to possess possibility for action (see The Social Partner’s Possibility for Action), and studies in which social partners did not appear to have possibility for action (see No Perceived Possibility for Action). At the most apparent level, this involved distinguishing between on-line and off-line, or primed social contexts. While the former included interactions in which the social partner was physically present, the latter used social stimuli (e.g., photographs) rather than interactions with a live social partner; thus, the two contexts differed in possibility for action by the social partner.

Secondly, independent of this “possibility for action” subdivision, we distinguished between different types of relationships, according to whether the social partner was a stranger (e.g., Jackson et al., 2005), a parent (e.g., Vervoort et al., 2011), a friend (e.g., McClelland and McCubbin, 2008), or the romantic partner (e.g., Master et al., 2009) of the person in pain (see Relationship Between the Social Partner and the Person in Pain). We further particularly examined studies according to the interaction history, contrasting rich interaction histories (parent, partner, and friend) with one-off interactions with strangers. Previous experiences with the social partner were considered to be important not only from an attachment perspective (see below) but also in terms of predictability of the other’s mental state, which we address in the discussion.

It is important to emphasize that while we organized the data around the categories of “possibility for action” and “type of relationship” separately, most aspects of interpersonal interactions are likely to interact in various dynamic ways to create the overall social context of pain. We point the reader to related sections as appropriate throughout the review and present an overall theoretical conceptualization (based on a free energy framework) of such dynamic patterns in the discussion.

### The social partner’s possibility for action

Twenty-two studies manipulated aspects of interpersonal interactions in which the social partner was physically present and was perceived to have the possibility to act toward the person in pain. In nine of these studies, the social manipulation involved the opportunity of engaging in verbal communication with a social partner, while the remaining 13 studies manipulated social presence without verbal communication.

#### Possibility for action and verbal interaction

In studies manipulating verbal interactions, the social partner was generally designed to act in a socially supportive role toward the person in pain. Social support is a complex construct, broadly conceptualized as “resources and interactions with others that help people cope with problems” (Masters et al., 2007, p. 11) and thus includes clear possibilities for action. Six studies (Chambers et al., 2002; Brown et al., 2003; Jackson et al., 2005, *studies 1 and 2*; Jackson et al., 2009; Wilson and Ruben, 2011) included conditions in which the content of the interaction was unstructured (i.e., not pre-determined by the experimenters). In Brown et al.’s (2003) study, this unstructured condition took the form of an “interaction” condition, in which the social partner was not instructed to behave in any particular way and the participant could shape the interaction. Three further conditions involved active support (explicitly supportive comments), passive support (presence without verbal interaction), and experiencing pain alone. Regardless of whether the social partner was a friend or a stranger, participants reported more pain in the unstructured interaction condition and when experiencing pain alone than they did in the active and passive support conditions (Brown et al., 2003). Though the authors did not record the verbal content of the unstructured interaction condition, they suggested that the interaction could have included negative comments, which may have counteracted any benefits of social support. Similarly, Jackson and colleagues found in a first study that participants who spoke to an empathic experimenter (“transaction opportunity” condition) showed reduced pain tolerance and increased pain intensity compared to participants who did not speak to the experimenter (Jackson et al., 2005, *study 1*). This was mirrored in their second study (Jackson et al., 2005, *study 2*), in which female participants displayed the lowest pain tolerance in the transaction opportunity condition, compared to structured conditions, such as distraction and encouragement conditions. Lastly, Chambers and colleagues trained mothers to respond to their children’s pain in either pain-promoting ways (which included reassurance, empathy, mild criticism, and giving control to the child) or pain-reducing ways (which included distraction with an alternative task, humor, and “encouragement to use coping strategies”), or mother and child interacted as normal in an unstructured way. Girls reported highest pain intensity in the pain-promoting group, followed by the unstructured interaction, and then the pain-reducing condition (Chambers et al., 2002). These effects were not present in boys, in accordance with other findings (Jackson et al., 2005, *study 2*; Jackson, 2007). As the pain-promoting condition included different social attitudes (e.g., empathy and mild criticism were included in the same interaction condition), it may have been more mixed than other structured conditions, in accordance with Brown et al.’s explanation regarding their unstructured condition. It thus seems that unstructured or mixed valence verbal interactions with an observer can worsen the experience of pain.

In addition, Wilson and Ruben (2011) discovered that adult attachment style moderated the relationship between unstructured verbal interactions and pain. Attachment styles derive from attachment theory (Bowlby, 1977) and are individual differences in interpersonal relating formed in infancy over repeated interactions with a primary caregiver. These styles are relatively stable across the lifespan (Main et al., 1985; Waters et al., 2000). In adulthood, attachment styles are generally classified as secure or insecure; the latter commonly being subdivided into anxious and avoidant styles (though attachment styles are also viewed dimensionally, e.g., Fraley et al., 2000). In brief, securely attached adults are typically comfortable with closeness and depending on others, while anxious adults are preoccupied with the relationship and fear abandonment, and avoidant individuals are uncomfortable with closeness and lack trust in relationship partners (Hazan and Shaver, 1987). Wilson and Ruben found that in couples in which the woman received noxious stimuli, highest pain was reported when both members of the couple had higher levels of attachment anxiety. Further, avoidant women showed lower pain tolerance when the social partner was more anxiously attached, whereas securely attached women showed higher pain tolerance when the social partner was more anxiously attached (Wilson and Ruben, 2011).

In a related study examining the moderating role of attachment style, Sambo et al. (2010) manipulated the “perceived empathy” of a present social partner. While empathy predicts social support provision (Devoldre et al., 2010) and plays an important role in health care settings (Blasi et al., 2001; Tait, 2008), its effects on pain remain understudied in experimental contexts, though some studies have included empathy as one element of a multifaceted manipulation (e.g., Chambers et al., 2002; see above). “Perceived empathy” describes participants’ knowledge of the social partner’s level of understanding of their pain (Sambo et al., 2010). In a within-subject design, Sambo et al. informed participants prior to the administration of noxious stimuli that each of the experimenters present during each of two identical blocks of noxious stimulation had either expressed high or low empathy for them during initial thresholding (determining a participant’s pain threshold). In a third condition, participants experienced pain alone. Although participant and social partner did not communicate during pain induction, the empathy manipulation was verbal, and is thus reviewed here. The perceived empathy manipulation interacted with the participants’ adult attachment style to affect pain, in that higher attachment anxiety predicted less pain in the high empathy compared to the low empathy condition. However, it should be noted that the manipulated facet of empathy was thus quite “cognitive” in nature and it is unclear how it may compare to natural social contexts in which empathy is not only verbally but also behaviorally communicated.

Moreover, four experiments manipulated structured interpersonal interactions, that is, instances of interpersonal interactions with set verbal elements (e.g., certain sentences the interaction partner always used) or a clear theme (e.g., supportive comments). Conditions such as distraction, reinterpretation, and encouragement (Jackson et al., 2005) and active support (Brown et al., 2003) all led to increased pain tolerance relative to transaction opportunity conditions (see above). Although Chambers et al. (2002) included several verbal elements, the nature of their pain-reducing condition was supportive overall, and indeed it was found to reduce pain relative to other conditions in girls. While these interactions all exerted pain-reducing effects, it seems likely that the mediating mechanisms may have differed, since the interactions compared were quite varied, e.g., distraction vs. reinterpreting pain-related cognitions (Jackson et al., 2005) (see Discussion).

In summary, unstructured verbal interactions were found to increase pain and were influenced by adult attachment style. The effects of structured verbal interactions depended on the content and valence of the interaction. While interactions with a clear theme positive valence, e.g., encouragement, reinterpretation, and active emotional support, reduced certain pain measures, interactions with mixed verbal content, and valence were pain-promoting, even when they included one of the above “positive” factors such as reassurance. Notably, the effects of verbal interactions on pain were mainly found in women but not men.

#### Possibility for action and non-verbal social presence

Thirteen studies manipulated social presence during pain without verbal communication. Manipulations ranged from the mere presence of a supportive other (e.g., Flor et al., 1995) to varying the threat of the social partner (e.g., Peeters and Vlaeyen, 2011) and interpersonal distance (Modic Stanke and Ivanec, 2010), to conditions using hand-holding (Master et al., 2009). While some manipulations did not involve any actual interaction between person in pain and observer, possibility for action by the social partner was salient as the social partner was physically present. Further, though several studies placed the social partner in an adjacent observation room (Kleck et al., 1976, *studies 1 and 2*; Vervoort et al., 2008, 2011), we considered that participants perceived their partners as capable of action because participants were aware that their social partner was observing them *during* pain induction (as opposed to encounters prior to pain induction, see Social Partner without Perceived Possibility for Action during Pain). In addition, the results of these studies were comparable with the other studies in which the social partner was present in the same room, in that social manipulations influenced participants’ facial expressions, which portray a communicative intent (e.g., Williams, 2002).

Kleck et al. (1976, *studies 1 and 2*) discovered that participants showed reduced facial expressions and reported less pain when they were observed than when they were alone; this was also found for physiological measures (Sambo et al., 2010), pain ratings in participants who received passive support from a friend or a stranger (Brown et al., 2003) or reported having low levels of social support in general (McClelland and McCubbin, 2008); for participants with high levels of self-reported everyday social support, pain ratings were higher in the presence of a friend than alone (McClelland and McCubbin, 2008). In addition, participants with a solicitous spouse showed a reduced pain threshold and tolerance in the presence of the spouse vs. alone (Flor et al., 1995). These latter findings fit within models of chronic pain positing that high social support may be positively reinforcing and ultimately lead to increased and prolonged pain (see Insights from Clinical Studies). Furthermore, in the aforementioned study on the role of perceived empathy on pain measures (Sambo et al., 2010), avoidant attachment was the only factor that moderated the relationship between social presence and pain report, such that higher attachment avoidance predicted more pain in the presence vs. alone condition, possibly because avoidant individuals prefer to cope on their own.

In addition to the above moderating factors, several studies which tested the communal coping model of pain catastrophizing (see Insights from Clinical Studies) reported that pain catastrophizing moderated the effects of presence on pain. Unfortunately, the direction of such effects varied between studies: only high pain catastrophizers (Sullivan et al., 2004) vs. only low pain catastrophizers (Vervoort et al., 2011) were found to exhibit facial expressions for a longer time period in the presence of a social partner than when alone. In addition, Vervoort et al. (2008) demonstrated that low-catastrophizing children displayed less pain when a stranger rather than their parent was present. It is possible that a stranger may be perceived as more threatening than a parent, leading to the inhibition of facial expressions. Indeed, three studies varied perceived threat during the social situation. They found that the presence of a stranger during a threatening situation (Jackson et al., 2009; Vlaeyen et al., 2009), as well as the threat appraisal of the strangers themselves (Peeters and Vlaeyen, 2011) led to attenuated facial expressions of pain (Vlaeyen et al., 2009; Peeters and Vlaeyen, 2011) and reduced pain tolerance (Jackson et al., 2009), i.e., increased pain sensations.

Lastly, we considered pain-modulatory effects of interpersonal distance between social partner/s and the person in pain. Interpersonal distance can modulate intimacy between people (Sussman and Rosenfeld, 1982), and violations of personal space can increase aversion of the social partner (Sussman and Rosenfeld, 1978). Conversely, a sense of safety and intimacy provided by a trusted other might be diminished if they are physically distant and unable to help. However, only one study directly investigated the effects of physical distance on pain (Modic Stanke and Ivanec, 2010). In this study, the social partner was positioned either 0.5 or 1.5 m from the person receiving pain. No effects of distance on pain were found. However, both social partner and participants in pain were female. Women have been found to maintain smaller interpersonal distances (i.e., choose to sit closer together) and do not see close distances as violations of space compared to men (Sussman and Rosenfeld, 1978, 1982; Holland et al., 2004). Therefore, at present, we cannot draw any conclusions on the effects of interpersonal distance on pain.

Overall, social presence differentially impacts pain according to individual differences of the person in pain or of the social partner. Participants reporting higher levels of everyday social support and higher attachment avoidance, as well as participants with a solicitous spouse, had worse pain outcomes when a social partner was present than when they were alone, while participants with low levels of everyday social support showed the opposite effects. Unfortunately, the direction of the moderating effect of pain catastrophizing remains unclear, while environmental threat seems to exacerbate pain.

Only one study coupled social presence with a direct action toward the participant (Master et al., 2009). In this study, hand-holding was employed as a form of social support. In different conditions in a within-subject design, female participants held the hand of their partner, or the hand of a stranger, or held an object. Reductions in pain unpleasantness were found when holding the hand of the romantic partner during pain compared to when holding a stranger’s hand or holding an object (these differences were not due to distraction, as participants’ reaction times to random computer-generated beeps did not differ across conditions). This finding is consistent with a study showing that unpleasantness ratings and neural responses to the threat of electric shocks were reduced when participants held their spouse’s hand as opposed to a stranger’s hand or no hand at all (Coan et al., 2006). Although the latter study assessed “threat of pain” and not pain *per se* (and hence was not included in Table 1), taken together both these studies suggest that holding the hand of the romantic partner can reduce pain-related unpleasantness. However, a few methodological issues deserve mention. Hand squeezing was not measured in either study and holding a stranger’s hand may be a somewhat unusual and potential socially uncomfortable condition. Further, a condition without touch (i.e., holding no hand at all) arguably differs from the other two in terms of multisensory integration.

In conclusion, verbal and non-verbal interpersonal interactions with perceived possibility for action were found to be pain-reducing only when specific verbal behaviors with positive intention, such as supportive comments, reinterpretation, and distraction, or non-verbal interactions with a clear positive social meaning (e.g., holding one’s partner’s hand) were manipulated and participants had low levels of self-reported social support and attachment avoidance. By contrast, more unstructured, emotionally negative, varied, or vague social manipulations led to increases in pain, either directly (e.g., presence conditions with unstructured verbal content) or in interaction with variables linked to the perception of threat and anxiety, such as catastrophizing and threat manipulations.

### No perceived possibility for action

Studies with no possibility for action were defined by the absence of a social partner during pain induction. Here, social manipulations were classified according to two sub-categories. In a first set of three studies, interpersonal variables were manipulated by priming. Second, two studies involved a partner who was present *before* pain induction but not *during* pain induction.

#### Primed interpersonal contexts

Three studies presented participants with photographs of their partner and either a stranger and an object (Master et al., 2009; Eisenberger et al., 2011) or an acquaintance (Younger et al., 2010). All studies discovered that viewing pictures of the partner reduced pain relative to viewing stranger/acquaintance and object pictures. While such effects might be explained by distraction or familiarity of the partner, two studies assessed distraction (see Possibility for Action and Non-Verbal Social Presence for details on Master et al., 2009) and Younger et al. (2010) included a word-association task condition. They also controlled for familiarity by comparing viewing pictures of a partner with viewing pictures of an equally attractive and familiar acquaintance. However, the other two studies cannot rule out possible familiarity effects (Master et al., 2009; Eisenberger et al., 2011) and Eisenberger et al. (2011) cannot exclude possible distraction effects.

Two of the three studies (Younger et al., 2010; Eisenberger et al., 2011) also employed functional neuroimaging techniques and reported neural activations during the different social conditions. Most notably, pain attenuation in the partner picture condition was positively linked to activation in areas associated with safety-signaling (the ventromedial prefrontal cortex; Eisenberger et al., 2011), and reward processing (e.g., the caudate head and nucleus accumbens; Younger et al., 2010). Based on this finding, two neurocognitive mechanisms potentially mediating the beneficial effects of viewing partner pictures were put forward. First, ventromedial prefrontal cortex activation was not only found during partner picture viewing but was further negatively correlated with both pain ratings and pain-related neural activity. Hence, it was claimed that viewing pictures of an attachment figure (i.e., the partner) signaled safety in the face of threat (pain), which contributed to pain attenuation (Eisenberger et al., 2011). The second proposed mediating mechanism concerned reward-related neural activation, which has previously been positively associated with intense love (Aron et al., 2005). In Younger et al.’s (2010) study, viewing pictures of the partner and a distraction task both reduced pain, but only the partner condition was associated with activation in the bilateral caudate head, bilateral nucleus accumbens, amygdala, hypothalamus, pregenual ACC, and medial orbitofrontal cortex, which play a role in the processing of rewards (Aron et al., 2005). Reward processing has in turn been linked to pain attenuation (e.g., Wood, 2006) and placebo analgesia (e.g., Scott et al., 2007). Such mechanisms may explain why pain was not attenuated when viewing pictures of strangers or acquaintances, who are not involved in a pre-existing loving attachment relationship with the person in pain.

Taken together, showing participants photographs of their romantic partner may reduce pain by priming attachment or love themes and related brain networks. The role of distraction and familiarity in some of these studies remains to be established.

#### Social partner without perceived possibility for action during pain

Two studies employed a social partner who interacted with the participant before but was absent during pain induction (Platow et al., 2007; Borsook and MacDonald, 2010). The first investigated whether the effects of reassuring comments depended on in-group or out-group status of the social partner, while Borsook and MacDonald studied socially induced hypoalgesia (reduced pain in the face of a stimulus that is normally perceived as painful; IASP, 1994) by negative vs. positive interpersonal interactions. Contrary to studies with perceived possibility for action (see The Social Partner’s Possibility for Action), both found that positive encounters *before* pain induction did not affect pain ratings or pain tolerance during pain induction, highlighting the importance of possibility for action. However, reassurance from an in-group member did selectively reduce physiological arousal (Platow et al., 2007). Furthermore, negative interpersonal interactions preceding pain induction were associated with reductions in pain ratings, attributed to social harm induced hypoalgesia (Borsook and MacDonald, 2010).

In sum, social manipulations characterized by the absence of a social partner during pain seemed to reduce pain only when they were interpersonally relevant, e.g., when there existed a close (attachment) bond between the social partner and the person in pain, or the social partner was an in-group member of the person in pain.

### Relationship between the social partner and the person in pain

The reviewed studies differed in terms of the relationship between participant and social partner. Most commonly, the social partner was a stranger (18 studies), the partner (six studies), a parent (three studies), or a friend/acquaintance (four studies) of the participant^1^. In one study, the nature of the social partner was not specifically defined (had to be at least an acquaintance but could also be the partner, Jackson et al., 2009; counted as acquaintance above), and thus it was not possible to evaluate relationship effects in this study.

Perhaps due to partner manipulation studies coming from similar research backgrounds and being designed to be positive (e.g., supportive), these studies generally found that the romantic partner reduced pain, although this effect was moderated by adult attachment style (Wilson and Ruben, 2011) and spouse solicitousness (Flor et al., 1995). Also, social manipulations were more homogeneous when the social partner was the romantic partner, presumably because they were constrained by the couple’s relationship history. For example, a very empathic partner being assigned to a high social threat condition in which they supposedly chose to administer a high number of pain trials to their partner (as in Peeters and Vlaeyen, 2011) might not seem believable to the person in pain. Likewise, certain social manipulations such as hand-holding may be inherently more suitable for pre-existing relationships.

The effects of interacting with strangers were most diverse, possibly due to the range of social manipulations, social meanings, and varying degrees of knowledge of the stranger’s mental state. For example, some studies gave participants no information about the stranger’s mental state (e.g., Sullivan et al., 2004, stated that the stranger was present only to monitor the water temperature of the coldpressor), some presented personally irrelevant information about the stranger’s mental state (e.g., Vervoort et al., 2008, informed participants that the stranger present was a student observing the experimental session to learn about the pain procedure) and others gave participants personally relevant information regarding the stranger’s mental state, for example how much empathy the stranger had for the participant (Sambo et al., 2010). When interactions with different types of social partners were compared within the same experiment, with social manipulations remaining constant, partners were found to reduce pain more than strangers or acquaintances (Master et al., 2009; Younger et al., 2010; Eisenberger et al., 2011), but most of these studies did not control for familiarity effects. Parents did not differ from strangers in their impact on pain intensity but increased facial expressions of pain relative to strangers in low-catastrophizing children (Vervoort et al., 2008). Furthermore, interacting with a friend was found not to differ from interacting with a stranger in affecting pain (Brown et al., 2003).

Overall, it appears that the type of relationship between participant and social partner may moderate how interpersonal factors affect pain. In general, pain seems to be attenuated when the participant is receptive to support (e.g., anxiously attached) and knows the partner is positively oriented toward them (but not highly solicitous), either due to a pre-existing relationship (e.g., between romantic partners), or experimental manipulation (e.g., empathy levels of previously unknown confederates are communicated to the participant). Comparing different types of relationships within the same experimental set-up to tease apart the relative influence of social manipulation and interpersonal relationship on participants’ pain remains an ongoing issue for future research.

## Discussion

This paper aimed to provide a systematic review of experimental studies investigating how interpersonal factors influence pain perception and communication. We examined 26 studies with a focus on the type of social manipulations, individual difference characteristics, and the person in pain’s relationship with the social partner. Overall, we found that unambiguously positive verbal and non-verbal interactions or positive interpersonally relevant primed interactions reduced pain, while negative, mixed valence, or ambiguous interactions led to increases in pain-related measures. These findings were moderated by individual differences of the person in pain and the social partner, such as adult attachment style.

We propose that the key findings from this review can be integrated into a free energy framework (see Introduction). Specifically, we argue that the perception of interpersonal interactions in the context of pain can affect perceptual and active inferential processes about pain by influencing the certainty or precision of an individual’s predictions about an impending stimulus vs. the certainty or precision of related prediction errors. Top-down predictions are not just about the content of lower level representations but also predict their context, defined in mathematical terminology as the *precision* of a probability distribution (inverse variance or uncertainty; Friston, 2009). Thus, precision refers to confidence in predictions; for example, the allocation of attention toward appropriately salient events can optimize the confidence in prediction errors and influence the relative weighting or importance of prediction errors (Feldman and Friston, 2010). This kind of top-down prediction in sensory cortices is thought to be mediated by cholinergic neuromodulatory mechanisms that optimize the attentional gain of populations encoding prediction errors (Feldman and Friston, 2010), as well as by dopamine in fronto-striatal circuits (Fiorillo et al., 2003). In interoception, precision may relate to attention to signals from the body or interoceptive sensitivity (Farb et al., 2013; Fotopoulou, 2013) and may be modulated by several contextual factors. Therefore, interpersonal interactions may affect pain by changing the precision of top-down predictions about pain. This notion of social modulation as precision modulation can be seen as similar to previous psychological accounts (e.g., Van Kleef, 2009) which put forward that social interactions inform inferential processing of the environment (e.g., in developmental research, a mother’s facial expressions may influence processing regarding the safety vs. threat of a visual cliff environment). Integrating such notions within a predictive coding model places them in a wider and neurobiologically plausible framework.

### Interpersonal interactions as precision modulation

Based on the reviewed studies, we put forward that interpersonal interactions may affect the precision of an individual’s predictions and thus pain in at least two ways: (a) by signaling the safety or threat of noxious stimuli (interoceptive salience) or (b) by signaling the safety or threat of the environment in which stimuli occur (environmental salience).

#### Precision of predictions about an impending stimulus itself

The present review revealed that certain interpersonal interactions may directly signal information about the safety or threat of an impending stimulus. Supportive interactions focusing on the painful sensations themselves increased pain tolerance, while interactions in which the threat of noxious stimuli was emphasized reduced pain tolerance. Specifically, a social partner helping participants to re-interpret uncomfortable sensations as neutral or positive sensations increased pain tolerance scores (Jackson et al., 2005) and decreased pain intensity ratings (Jackson, 2007). The social partner signaling that the noxious stimulus was safe thus shaped participants’ prediction that the stimulus was safe, which in turn may have reduced the salience of the stimulus and thus pain. In contrast, both social partner and participant reading threatening information about the noxious stimulus increased the number of pain words in their conversation during pain and decreased pain tolerance relative to other conditions (Jackson et al., 2009), possibly because the social partner amplified the threat and hence improved precision and salience.

Moreover, the present results showed that interpersonal interactions may influence the salience of noxious stimuli by modulating the participant’s attention toward or away from the noxious stimulus. Verbal interactions directing the participant’s attention away from the noxious stimulus (e.g., Chambers et al., 2002; Jackson, 2007), non-social distraction conditions (Younger et al., 2010), and conditions in which distraction could have been a factor (Eisenberger et al., 2011) generally found that diverting attention away from the noxious stimulus led to increased pain tolerance and reduced pain ratings. Therefore, distraction might attenuate pain by reducing the precision of top-down predictions, which in turn may have decreased the salience of the noxious stimulus and hence pain.

#### Precision of predictions about the environment

The reviewed studies highlighted that in addition to information about the impending stimulus itself, interpersonal interactions may signal safety or threat of the environment in which the stimulus will occur, and thus modulate pain. In particular, interactions with a clear content regarding the provision of safety or support, or the partner having the possibility to act to protect the person in pain might increase the perception of environmental safety and thus indirectly decrease the perceived threat of noxious stimuli. Indeed, explicitly supportive verbal (e.g., Brown et al., 2003) and embodied (hand-holding; Master et al., 2009) interactions reduced pain, while pain-promoting and threatening conditions increased pain (Chambers et al., 2002; Jackson et al., 2009). By contrast, interactions without a clearly supportive content or possibility of supportive action may not increase the safety value of the environment. Indeed, the present review revealed that unstructured or mixed verbal interactions led to more pain relative to structured verbal interactions with supportive content.

### The perception of interpersonal interactions *per se*

In addition to the influence of interpersonal interactions on pain, the perception of interpersonal interactions themselves may depend on (a) an individual’s prior beliefs about interpersonal relating and the meaning of related interactions, and (b) the certainty or precision with which an interpersonal interaction may predict environmental threat or safety.

#### Prior beliefs about interpersonal relating

When examining the effects of interpersonal interactions on pain, it is vital to take into account “historicity”; that is, the back-drop of individual social development against which current social exchanges are placed (Schilbach et al., in press). In a free energy framework, such consideration entails examining not only how predictions are updated “on-line,” but also across the life span in slower time scales (Friston, 2009). In the reviewed studies (as well as in clinical studies on pain, see Insights from Clinical Studies), several individual characteristics were found to play a role in the perception of interpersonal interactions and how they influence pain. While the role of factors such as catastrophizing and gender remains unclear, the application of attachment theory in pain research has generated some convincing results. Attachment theory posits that from early in life, attachment figures can serve as a “secure base” from which the infant explores the world (Bowlby, 1977). If a secure attachment bond is formed over repeated instances of responsive caregiving, the “secure base” signals safety to the infant, while insecure bonds lead to more ambivalent or even threatening signals from others. These bonds lead to the formation of attachment styles, which remain relatively stable into adulthood (see also section Possibility for Action and Verbal Interaction). In the clinical pain literature, insecure attachment styles have been proposed as crucial vulnerability factors for developing chronic pain (Meredith et al., 2008).

In the reviewed experimental studies, differences in attachment style influenced the effects of interpersonal variables on pain. Sambo et al. (2010) found that participants characterized by higher attachment anxiety, i.e., a fear of abandonment and need for reassurance from others (Mikulincer et al., 2009), reported less pain when social partners showed high compared to low empathy. Hence, when a partner was ostensibly positively oriented toward the participant, and the participant’s attachment style was characterized by seeking for signs of reassurance, the social partner signaled safety to the participant, which in turn may have led to the reduction in pain. In contrast, pain was increased when both members of a couple were highly anxious relative to other attachment style constellations (Wilson and Ruben, 2011), possibly because the partner was not able to signal the desired reassurance. Similarly, avoidant women showed lower tolerance when their partner was highly anxious and higher tolerance when their partner was low anxious, reflecting the detrimental effects of environmental anxiety cues on pain. More generally, avoidant individuals, who generally have low trust in others, reported more pain when with others than when alone (Sambo et al., 2010). Overall, the findings highlight the importance of attachment priors in affecting the perception of the social partner.

#### Precision of the salience of the social partner

In addition to prior beliefs about interpersonal relating, we found that the specificity or salience (precision, mathematically, see above) by which an interpersonal interaction may predict environmental threat or safety might influence how interpersonal interactions are perceived and ultimately how they affect pain. Relevant factors are (1) the transparency of the social partner’s intentions and thoughts, (2) the social partner’s possibility for action, and (3) the familiarity or the degree of social bonding with the social partner.

Firstly, knowledge of the social partner’s mental state might determine the salience of a social interaction. Information provided in the experiment (e.g., the social partner’s empathy toward the participant) and prior knowledge about the social partner’s mental state and intentions may increase precision, while lack of knowledge of the social partner’s mental state may have the opposite effects. Thus, “pure” presence conditions yielded mixed results (see Possibility for Action and Non-Verbal Social Presence), possibly due to lack of information about the intentions, and thoughts of the social partner. Interestingly, unstructured and mixed interactions (which mostly occurred with strangers) were found to increase pain (see Possibility for Action and Verbal Interaction), indicating that uncertain interpersonal interactions do not weaken the impact on pain but rather may even signal increased environmental threat.

Secondly, a social partner’s possibility for action may influence the salience of interpersonal interactions. Specifically, the reviewed studies revealed that interpersonal interactions without possibility for action during pain did not affect pain as much as interactions with possibility for action (see Social Partner Without Perceived Possibility for Action During Pain). Exceptions included interactions that were also interpersonally relevant, in which case other mechanisms may have enhanced salience. From the free energy perspective, these findings can be understood as active inference “by proxy.” Normally, action minimizes prediction error by changing sensory input (Friston, 2009). In the case of pain in the presence of others who possess possibility for action, social partners may represent an auxiliary action system; they are able to act to change the sensory input for the person in pain (e.g., by pulling a person’s hand away from a noxious source). Within an experimental context, the social partner cannot usually change a participant’s sensory input. However, they can possess the possibility to do so, e.g., by being present in the experimental setting. Therefore, the higher the perceived possibility for action, the higher the salience of the interaction in terms of influencing safety and threat. Unfortunately, to our knowledge, no study has specifically examined the effects of partners’ actual actions on noxious stimuli and therefore this facet of the interpersonal modulation of pain requires further experimental exploration.

Thirdly, interacting with a familiar social partner might also enhance the salience of the social interaction. Close bonding and positive relationship histories (e.g., secure attachment relationships) with established trust may lead to precise predictions of environmental safety in interpersonal interactions with the romantic partner (e.g., Eisenberger et al., 2011). Indeed, our review brought out that positive interactions with romantic partners generally reduced pain measures, except when partners were overly solicitous or insecurely attached (Flor et al., 1995; Wilson and Ruben, 2011). These findings extended to paradigms where the partner was not physically present, but related cognitions and feelings were primed in the individual in pain (e.g., Younger et al., 2010). On the contrary, interactions with strangers yielded mixed results (Jackson et al., 2005; Vlaeyen et al., 2009). Moreover, one study observed that greater relationship quality and bonding between partners was associated with greater pain reduction when photographs of the partner were shown during pain (Eisenberger et al., 2011).

### Overview of effects and the overall framework

In summary, we found that clear and structured experimental interactions may lead to reductions in pain measures when they signal safety of the noxious stimulus or the environment in which it occurs or they are designed to direct attention away from the noxious stimulus. These effects are particularly apparent when the interpersonal interaction itself is salient. However, in most cases, the beneficial effects of support will be moderated by characteristics of the person in pain, such as their attachment style and level of pain catastrophizing. Although more data is needed and some studies found contrary effects, the general trend thus far is that insecure attachment and catastrophizing coping strategies worsen the pain experience, particularly during interpersonal interactions that may be ambiguous.

**Figure 1:**
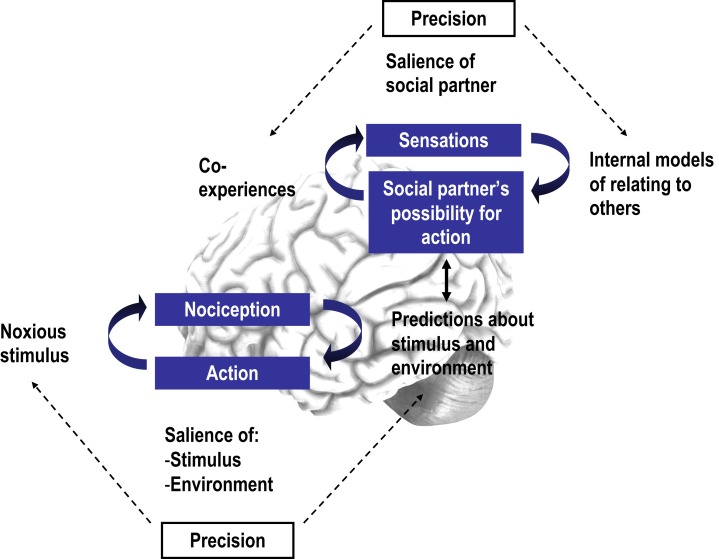
**A schematic representation of our free energy framework**. The bottom panel depicts how interpersonal interactions may modulate the precision of interoceptive predictions, while the top panel shows the perception of interpersonal interactions *per se*, and how these influence interoceptive predictions in a top-down manner; the arrow is two-headed to show the interactive nature of the two hierarchical levels. Precision arrows are dashed to demonstrate the dynamic and modulatory, rather than permanent, influence of social context.

We have put forward a free energy framework for integrating these findings in a unified, biologically plausible theoretical framework. Our key proposal is that the perception of salient interpersonal interactions may enhance *the precision* of predictive signals regarding the salience of a noxious stimulus in a given environment, thus ultimately affecting the perceptual and active inferential processes that lead to pain perception and related motivated actions. Specifically, interpersonal exchanges affect precision or salience by socially signaling the safety or threat of the impending stimulus itself or the environment in which the stimulus occurs. In turn, at higher levels of the neurocognitive hierarchy and at slower time scales, the perception and interpretation of such interpersonal variables themselves may depend on an individual’s prior beliefs about interpersonal relating and the certainty by which an interpersonal interaction may predict environmental threat or safety. A schematic overview is presented in Figure 1.

The precise neurobiological mechanisms by which interpersonal interactions affect pain remain to be determined. Initial findings suggest that their precision-based modulatory role in pain may be related to dopamine-based motivational mechanisms that have been implicated in the rewarding and craving aspects of social bonding in both humans (Younger et al., 2010) and animals and/or their co-activation with opioid and oxytocin mechanisms (see Insights from Animal Studies and Implications for Human Research). Oxytocin is a neuropeptide that has been implicated in social bonding (e.g., Strathearn et al., 2009), attachment (Buchheim et al., 2009), the social modulation of stress (Heinrichs et al., 2003; Chen et al., 2011), and has been shown to increase the salience of social stimuli (see Bartz et al., 2011 for a review), perhaps to simultaneously reduce the salience of bodily threat during social experiences that may be valuable for survival (e.g., reproduction, birth etc.). Thus, future studies could explore the role of such neuromodulatory mechanisms and their interactions during pain in social contexts.

### Limitations

While this paper represents the first systematic review of the social modulation of experimentally induced pain literature, a meta-analysis including direct, quantitative comparisons between studies was not possible due to the great methodological heterogeneity between studies. We were also not able to sufficiently address aspects of study quality, such as sample size, which differed across studies and may have explained some of the variation in findings. A further methodological difference of importance that we could not include was the diversity in study designs, such as sampling issues, ecological validity, pain induction methods, and type of pain measures obtained. For example, interpersonal interactions seem to have differential effects depending on the aspect of pain measured (e.g., pain catastrophizing studies in Possibility for Action and Non-Verbal Social Presence). As most studies included a subset of pain measures, it was not always possible to draw firm conclusions regarding the dependency of the findings on the specific pain measure used. It is further well-recognized that despite the potential of experimental studies to establish causality, the complexity of interpersonal interactions cannot be adequately operationalized in lab-based studies. Similarly, conclusions reached from studies on experimentally induced pain cannot be directly generalized to clinical pain due to the unique environmental and biological characteristics of the latter. Lastly, although we teased apart the different elements of the studies reviewed here to clarify their individual influences, many studies included composite elements within a single manipulation and further research is needed to determine the relative importance and weighting of these factors.

### Future perspectives and challenges

Although our review focused on interpersonal interactions between a (pain-free) social partner and a person receiving experimentally induced pain, the inclusion of other branches of research, such as social modeling studies and studies manipulating intergroup variables may provide a complementary picture to the present review.

Regarding the design of future studies, more specific manipulations focusing on certain aspects, for example safety or threat or particular facets of social support, could be employed and attempts made to replicate findings with previously used social manipulations. Furthermore, we suggest that varying perceived possibility for action, for example by using both live and primed interactions, and employing different kinds of social partners within the same experimental context (keeping the social manipulation constant) would be interesting avenues to explore. Individual differences, such as attachment style and pain catastrophizing, should also be taken into account in future studies.

Expanding on the proposed predictive coding framework, including several instances of interaction and measuring updating of safety vs. threat would be interesting, as well as investigating how lack of precision in interpersonal interactions impacts pain. Nevertheless, focusing on other mechanisms such as reward, attention/distraction by partner, and emotion regulation (e.g., the social baseline model; Coan, 2011) would also be important. Future neuroimaging studies focusing on safety- and threat-related neural activation during corresponding interpersonal interactions (e.g., Coan et al., 2006; see Vrticka and Vuilleumier, 2012, for a review related to attachment style) may add valuable insights into the neural mechanisms of the social modulation of pain. Finally, it could prove fruitful to study the central role of the neuropeptide oxytocin in parallel with manipulations of the interpersonal modulation of pain.

## Conflict of Interest Statement

The authors declare that the research was conducted in the absence of any commercial or financial relationships that could be construed as a potential conflict of interest.

## Footnotes

^1^Note that the total number exceeds 26 because several studies included more than one type of social partner.
